# Merging academy and healthcare in the Public Health training of medical students

**DOI:** 10.1186/s40985-020-00146-1

**Published:** 2020-12-03

**Authors:** Teresa Leão, Henrique Barros

**Affiliations:** 1 EPI Unit, Instituto de Saúde Pública da Universidade do Porto (ISPUP), Porto, Portugal; 2 Departamento de Ciências da Saúde Pública e Forenses e Educação Médica, Faculdade de Medicina, Universidade do Porto, Porto, Portugal

**Keywords:** Medical education, Public health practice, Competency-based education, Public health

## Abstract

**Background:**

Public Health remains central to understand health and its determinants, and Public Health teams are essential for an integrated collaborative medical practice. However, current teaching of public health to medical students varies in the European Region though an investment in multidisciplinary workforce is recognised essential to deliver high quality public health services. A recent medical education curricula restructuring in the University of Porto Medical School resulted in the inclusion of a Public Health module linking academic teaching to field practice and provided the opportunity to make an initial appraisal of students’ perceptions.

**Case study:**

We analysed final reports (*n* = 196), debriefing meetings notes (*n* = 2), and e-mails sent by students (*n* = 34) regarding the activities they observed or participated at, their contact with Public Health services’ teams, knowledge and critical appraisal, and opinion about the module. Students gained basic knowledge about how epidemiological surveillance, environmental health, health planning, and health promotion are performed in practice. They reported a better understanding of the roles and importance of Public Health services and its teams. Most considered that this module had an important role in their training. Some activities observed in the field lacked the needed standardisation to provide the students the feeling that core operations were experienced, which needs to be addressed in the future.

**Conclusions:**

Public Health practice-based training within field institutions may bring a better understanding of the discipline and specialty for medical students. It may strengthen interconnectivity and coordination of healthcare agents, which may improve future medical practice with potential improvement of patient-centred care and in terms of public health response, and back their roles as health agents and decision-makers.

## Background

To work in healthcare, in the present, is to be daily confronted with new challenges. Many of them were thought to belong to the past, as life conditions would have improved, or naively believed to be solved by the development of technology [1, 2]. In some cases, the prevalent opinion was a sort of urban myth [3]. Indeed, if health technologies contributed to increase the life expectancy of most people with chronic diseases in the high-income countries, it did not solve it all. Almost as a *volte face*, the development of technology and its ubiquitous presence in society contributed to the return of some infectious diseases and their reinstalling in countries they were absent for more than half a century, to the emergence of antimicrobial resistance with little organised reaction, and to expose most of the world’s population to pollution, highly processed food and drinks, or to sedentarism.

Few decades ago medical students did not put too much effort on memorising the aetiology, physiopathology or medical treatment of distant diseases such as dengue or Ebola. They believed drugs would solve most consequences of unhealthy behaviours, which patients stubbornly keep. Though, after being absent from the European Union since the 1920s, autochthonous cases of dengue were reported in Madeira in 2012 [4], and again in France and Spain in the last year [5]. The fear of pandemics continued substantial, first with Ebola [6], and in January 2020 with the SARS-CoV-2 epidemics [6]. Infectious diseases are disrespectful of borders and the high mobility of the population accelerates their dissemination, independently of the countries’ income. Climate change creates the perfect context for vectors to survive even during winter months [7]. Cumulatively, non-communicable diseases’ burden is raising even in the most deprived countries, creating *improbable duets* of raising obesity trends in countries where children still die of malnutrition (including undernutrition) [8, 9]. Multinational brands supply highly processed food and tobacco in a global platform, independently of race, language or culture differences, disseminating as microbes have since the beginning of times.

Most educational programmes designed to prepare health care professionals were not organised to accommodate this rapid often unpredictable changes [10, 11]. As knowledge in biology, chemistry, and genetics grew, these disciplines were added to the traditional anatomy-centred curriculum and extended the—much needed—understanding of physiology and pharmacology. Students became proficient on zooming into the micro dynamics of the disease and its treatments, with the risk of losing their ability to see the context of the patient or to understand the reasons behind their unhealthy behaviours. However, health care professionals must be caregivers, communicators and educators, team members, managers, leaders, and policy-makers. They must be able to link people to technology, information, and knowledge. As knowledge brokers, health workers are the human faces of the health system [11].

Multiple curricular experiences during the last four decades tried to respond to a changing global world, a changing social paradigm and of course a demographic, epidemiological, and nutritional transition that resulted in a changing case-mix to be confronted with. Some were in-depth revisits to longstanding hallmark curricula, as was the case of “The Johns Hopkins University School of Medicine curriculum for the Twenty-first century” [12] proposal. It responded to the one-century-old challenge of Flexner, who wrote what probably continues a central concern “It is indeed no paradox to assert that though medicine can be learned, it cannot be taught. The student must throw himself eagerly and intelligently into the quest. He must want to learn” [12]. This continuous need to learn and update was accelerated by the growing demand of patients and organisations for a more evident presence in decision-making at all levels—from drugs prescription to services organisation. The AIDS epidemic was pivotal in this shift. It resulted in a larger presence of societal aspects on education and training of medical students and young doctors, as did the Johns Hopkins module *Physician and Society*, and an additional need of stimulating students to become lifelong learners.

Flexner and Welch-Rose reports, in the first half of the twentieth century, had a major impact in health education and training in North America and worldwide. They all recommended to integrate modern biomedical, social, and behavioural sciences into the core curriculum, and to link education to research in a university environment. Flexner report introduced the modern sciences, as chemistry, physics and biology, and research as foundational for the medical curriculum creating the conditions for the birth of academic medical centers. Welch-Rose report provided competing visions of public health professional education: Rose favoured a national system of public health training with central national schools and a network of state schools, with emphasis in public health practice; Welch called for institutes of hygiene, following the German model, with increased emphasis on scientific research and connections to a medical school in comprehensive universities. Most schools of public health in the USA followed the Welch model as independent faculties in universities while both options co-exist to this day in Europe.

In Portugal, the academic training of medical students and professionals in public health remained in medical schools under the auspices of hygiene institutes until the sixties. A National School of Public Health was created in Lisbon and was in charge of academic training in public health of medical doctors, under the responsibility of the Ministry of Health and outside a university connection. Very recently (2006), in the University of Porto, an Institute of Public Health was created with the mission of providing multidisciplinary research and training in public health and public health sciences. However, Public Health training was independent of these institutes and medical schools remained the sole responsible for medical students’ curricula and education, and mostly disconnected from the field public health professionals.

Competing with a large and diverse number of disciplines for the students’ interests, resulting in limited resources offered to public health, academic and practice public health institutions need to build sustainable collaborations to foster teaching and practice-based research. As experienced in the USA, the academic health department concept offered a solution with joint leadership, multicomponent agreements, and shared professional development missions. In Portugal, the concept of Clinical Academic Centers was recently legally established bringing together health care institutions, research centers, and medical schools but left public health services away which precluded the leverage of resources and the promotion of sustainable collaborations.

Curricula are closely linked to historical legacy that codifies the traditions, priorities, and values of the faculty. It rarely is re-examined except for small changes, mostly accommodating new information or professional developments. The Faculty of Medicine of the University of Porto recently restructured its curricula. As a broader perspective in health became needed, stimulating the epidemiological thinking, linking health to its determinants, showing the existence of an unequal distribution of health across socioeconomic groups, and the importance of population level and intersectoral strategies to improve health and reduce inequities, Public Health-related modules were included but not explicitly placed under the “umbrella” of Public Health science. Populations’ health exposes first-year medical students to the theoretical concepts of morbidity and mortality, transmission and control of infectious diseases, as well as of chronic non-communicable diseases, and of health inequities, and Epidemiology and Clinical Epidemiology are included in the following year’s curricula. As knowledge must be materialised in practice [13], a module of Public Health was included in the sixth and last year of medical education. It is a module that aims at linking the theoretical teaching to a community and practice-based training. After a year and half of evolution, we analyse this public health module results and opportunities for improvement.

## Case presentation

### The public health module

It is constituted by three parts: (i) a one-day seminar that presents students how health planning and epidemiological surveillance are performed in the field, how environmental conditions are monitored and improved at the local level, how international health can protect the local populations’ health, and the role of Public Health consultants and of health authority; (ii) followed by three-week clerkship in Public Health Units during which students must write a report about the clerkship itself, critically describing the activities they observed or performed during the clerkship, and analysing them using Public Health evidence; (iii) a debriefing discussion on lessons learned and points to be improved.

In Portugal, public health services are constituted by fixed teams that aggregate a small number of medical doctors specialised in Public Health, nurses specialised in Community Health and Public Health, and Environmental Health officers; nutritionists, psychologists, or dentists can also integrate these teams but belong to a stock of common specialised resources. Small groups of students are distributed to each unit, where they are formally oriented by a Public Health consultant, the tutor, who must integrate the students and allocate them to field activities, make the link between what students observe and what would be the blueprint practice, show the Public Health evidence that supports it, and evaluate the students at the end of the clerkship. The program defines that students must perform field activities that, according to the availability of the unit, may vary between health promotion projects, environmental health activities, or epidemiological surveillance investigations, health observatory activities and health planning processes. The students may observe travel medicine consultations or ship inspection activities integrated in the application of the International Health Regulations, or other health authority activities as health inspections or physical incapacity evaluations. Journal clubs, with themes related to the practice of Public Health, are organised in some units.

### Achievements and pitfalls

We qualitatively analysed 196 final reports written during the last academic year and in the first semester of the current one, notes from two debriefing meetings and 34 e-mails with feedback from students, using thematic analysis with a deductive-inductive approach. Four themes were identified: (i) what activities students observed or participated at, (ii) their integration within the teams, (iii) how they used the knowledge gained in the description of the activities and on the critical appraisal, and (iv) their overall opinion on the module. In Tables 1, 2, and 3, we expose some of these students’ opinions regarding the activities they participated at, the integration within the teams, and their overall opinion about the module.

**Table 1:** Activities students observed or participated at during clerkship

*I believe the internship lacked practical [field] activities, and it would be more useful if [module’s] topics were taught closer the community and exercising the different practical methods used by Public Health consultants.* (Student 1) *The biggest disadvantage of the clerkship in this Unit was the small number of areas the students contacted with, as areas as health planning, environmental health and international health were not experienced.* (Student 2) *The tools used by these professionals, as outbreak simulations and the reflection on the public works in the city were stimulating for the learning process.* (Student 3)

**Table 2:** Integration of students within Public Health teams

*In the Public Health Unit I’ve met an interested team, attentive to the students. I’ve been well received and found an huge availability to answer to any doubts.* (Student 4) *I was able to take advantage of the multidisciplinarity of this specialty, being integrated in the Unit everyday activities.* (Student 5)

**Table 3:** Overall opinion of students about the module and, more specifically, clerkship, and seminars

**Module:** *[it] opened my horizons and allowed me to see Medicine with a widened perspective, contributing to, more the development as a future [health] professional, to my personal development, as it gave me critical sense regarding Health of the community I’m part of.* (Student 6) **Clerkship:** *this clerkship allowed me to contact directly with the daily life of a Public Health Unit, understanding what are its competences and specific functions which, in the future, even if I don’t specialize in Public Health, will be important do know when necessary to act.* (Student 7) *I found the clerkship quite interesting and with possible impact on the choice of the medical specialty of some medical students.* (Student 8) **Seminars:** *A positive note can be given to the first day of seminars in ISPUP, as concepts previously learnt were reviewed, as they are essential to go to the fieldwork, as well as to draw clear objectives and have a clear perspective on the Public Health clerkship.* (Student 9) *Though, I think that the time spent in this day was excessive. This limited the capacity to follow the focus of the presentations, not allowing us to have, by the end of the day, a clear view on the main ideas.* (Student 10)	

The activities students have participated at varied from unit to unit. The largest majority of students participated in environmental health activities and observed epidemiological surveillance investigations. Many assisted to health planning meetings and health observatory activities, and some to health promotion activities. They rarely assisted to ship inspection activities. Though not directly linked to the Public Health purpose but legally defined as a responsibility of the Public Health Authority in Portugal, many students contacted to the evaluation of patients’ physical incapacity. Many also assisted to travel medicine consultations.

The process of allocating students to Public Health Units presented several difficulties: these services have a small number of professionals (and the largest majority is sub dimensioned), they have limited physical conditions and, as such, most were able to receive two to four students per rotation. Furthermore, as medical students’ clerkship coincided with early career registrars’, nurses, and environmental health clerkships, fewer activities were available for them to observe and participate at (Table 1). Though students were presented to the whole team in most Public Health Units (Table 2), most were supervised by Public Health registrars and followed environmental health officers.

The analysis of the report of the clerkship, specifically the activities students participated at and their critical analysis showed that most students understood the basic concepts of Public Health and the functions of Public Health as a specialty. This was backed by the clerkship tutors in the students’ evaluation results. Though, only a few students were able to critically analyse the activities they observed in terms of effectiveness at the population level, of eventual adverse effects, or regarding costs and cost-effectiveness.

We encouraged students to share their perspective about the module, both in the final report, by e-mail and in the final debriefing. They stated that the module allowed them to understand the importance of the Public Health workforce, their functions, and/or, more specifically, the role of Public health consultants (Table 3). Many reported that this module brought a widening on their perspective of health. A few reported that after the module they considered Public Health as a career. Most reported that the module was an important gain in their education.

However, students also raised several issues. All noted that the first seminars’ day was too long and demanding (Table 3). They understood the need to concentrate all seminars before starting the clerkship in the units, and the need that this was compacted into 1 day so that it does not disturb the unit’s activities planning, but in the first year they noted that some seminars could be combined and others shortened. These suggestions were taken into account during the second-year module’s remodelling.

Regarding the clerkship, students referred they were not able to participate in the expected number or within the diversity of field activities (Table 1), and, in some cases, they were exposed to theoretical presentations that overlapped with the seminars, which they considered unnecessary. Many noted that the experiences largely differed across units, as the activities they observed varied between units and rotations. They suggested that problem-solving exercises in classroom would create a homogenous basis for all of them and should be included.

Feedback from Public Health Units, collected in a final meeting by the end of the first year and in informal conversations, showed that the largest majority of the tutors considered a positive experience to educate medical students. Though, as they lack the time to orient them and consider this an important experience to Public Health registrars, they proposed them as tutors. Public Health consultants and registrars frequently reported that organising the clerkship was difficult, and noted the difficulty of evaluating students’ knowledge. Public Health consultants and registrars seemed to value this experience in terms of career and training, as many asked for formal documents to the faculty certifying their participation as tutors.

## Discussion and conclusion

The overall positive feedback of the students, the clear positive evolution since the first-day seminar until their final reports, makes us believe that this scheme of practice-based learning integrated in those units responsible for the community-level implementation of Public Health activities could largely benefit the medical faculty curriculum.

Exposing medical students to Public Health Units increased their knowledge about surveillance and control of communicable diseases, of environmental conditions, and of the modifiable determinants of health. As Gillam et al. noted (2015) [14], this knowledge is “crucial in protecting the populations’ health” not only on the education of Public Health professionals but also of clinical doctors as they should understand the importance of acting in all levels of health prevention (primordial, primary, secondary, and tertiary prevention) and know how they are implemented in the real world.

Despite part of this knowledge could be transmitted through classroom simulation exercises and problem-solving, an expected positive effect of this module was to show students who are the flesh-and-bones professionals, what they do and, potentially, to allow students to see them as role models. This contact allows clinical and community-level professional groups to strengthen the network that should work coordinated during outbreaks’ investigation and control, during the design of health promotion programmes, or during healthcare planning challenges. For future clinicians, this will help them remember that although medical skills derive from the intrinsic aim of medicine, health care is delivered by teams. They learn that openness and flexibility are features of the educated rather than the narrowly trained mind and that the appreciation of activities valuable for their own sakes can provide a lifelong interest [15].

The contact with the community leads students to physically experience part of what they have learnt during the medical education regarding the determinants of health, observe people’s context, read the motives behind their unhealthy behaviours, and perceive why these are transmitted from generation to generation, from neighbour to neighbour. The comprehension of the determinants of health and health inequities may create a more humanistic approach to health and diseases, which is fundamental for the clinical practice and for their role as advocates for health and social policies [13]. Gaining knowledge on health planning and health promotion may be useful for critical thinking regarding planning and organisation of health programmes and services, decision-making, and participation as advocates for health policies and health reforms [13, 14]. Finally, the contact with the specialty opens their perspectives in health careers, looking beyond the hospitals’ walls into community health or healthcare management positions, and from the healthcare sector to non-governmental organisations or research organisations, amongst others.

Though, we must be aware of some limitations. First, there must be a higher balance between theoretical content in seminars and the practice during the clerkship. Indeed, students noted in the first year the long duration of seminars and overlap of some of those. These issues have been revised in the end of the first year and the number of students complaining decreased, but are still high. Considering that there is a moderate variation of the kind of activities students are exposed to, and as practice favours the knowledge acquisition [13], the next year module programme will probably include simulations and problem-solving exercises combined with online contents, as suggested by Godfrey et al. (2019) [16]. Second, students have a limited contact with Public Health consultants, despite our efforts. A major concern results from the fact that field public health has for years been away from academic activities and not formally collaborating with the university. This circumstance has also been noted by Lyon et al. (2015) [10] and is due to the small number of Public Health consultants, the sub dimensioning of the Public Health Units for the large number of needs raised by the communities, especially in terms of the health authority functions. An additional opportunity to envisage is the option for international clerkships, particularly considering the Community of Portuguese-Speaking Countries (CPLP). CPLP has formulated a strategic plan to improve health systems in all affiliated countries for universal access to high-quality health services. This includes the training of personnel and a network of projects to strengthen institutional capacity. These structures may provide medical training in largely diverse contexts, which may be appreciated by some students. Third, we used a regular feedback-based approach for the organisation of this module. Though, it may be biased as students could fear that their negative feedback could affect their final evaluation. However, we believe this bias may be small as, in the first year, students were evaluated exclusively by units’ tutors, and the suggestions were reported by e-mail with the teachers responsible for the module organisation. These suggestions also did not significantly differ from the first to the second year. The feedback given in their reports, by e-mail and in final meetings was also similar to the feedback given by a (very small) sample in formal faculty modules’ evaluation.

Considering the gains and these limitations, especially the fact that some students do not participate in core public health activities and that the contact with Public Health consultants tends to be short, which may limit their learning as a competency-based approach in Public Health, we understood that we must have a more active role in designing and stimulating the students learning process. This module’s curriculum must be tailored to offer the needed training for the today’s population health problems. Methods, as simulations, problem-solving exercises, and group discussions, may allow us to include these issues while maintaining flexibility to include emerging problems, and may also contribute to better assess the achievements and shortfalls of students in a more individualised learning process, trying to overcome the traditional one-size-fits-all approach.

As a conclusion, on the one hand, it seems clear that this field-based module may benefit the medical curricula and provide a tailored competences’ approach. On the other hand, we believe that the inclusion of Public Health consultants and registrars, nurses, and environmental health officials in medical education encourages them to acquire training competencies, and to analyse, improve, and value what they do. We expect this cooperation will bring a higher interconnectivity and coordination of the healthcare and society agents, and a clearer understand of the discipline and recognition of the specialty, which are increasingly needed in the medical action. This teaching project now presented will finally succeed if the partnership under construction evolves with increasing shared decision-making and management, based on explicit written agreements, respected expectations, and the continued commitment of the concerned partners: students, teachers, and practitioners.

## Data Availability

All materials supporting the conclusions of this article are available upon request.
