# Corrigendum: Merging Academy and Healthcare in the Public Health Training of Medical Students

**DOI:** 10.3389/phrs.2024.1607564

**Published:** 2024-06-07

**Authors:** Teresa Leão, Henrique Barros

**Affiliations:** ^1^ EPI Unit, Instituto de Saúde Pública da Universidade do Porto (ISPUP), Porto, Portugal; ^2^ Departamento de Ciências da Saúde Pública e Forenses e Educação Médica, Faculdade de Medicina, Universidade do Porto, Porto, Portugal

**Keywords:** medical education, public health practice, competency-based education, public health, Pregraduate education

The authors neglected to include the funder FCT - Fundação para a Ciência e Tecnologia, I.P., UIDB/04750/2020 and DOI identifiers https://doi.org/10.54499/UIDB/04750/2020.

The authors apologize for this error and state that this does not change the scientific conclusions of the article in any way.

